# Dietetic Regime in Cardiovascular Affections

**Published:** 1913-11

**Authors:** 


DIETETIC REGIME IN CARDIOVASCULAR AFFECTIONS. H. Vaquez, Paris, *Arch. des Mal. du Coeur, des Vaisseaux, et du Sang.*, 1913, VI, 225. Vaquez contributes a comprehensive paper on diet in diseases of the circulatory apparatus. Concerning arteriosclerosis he points out that there exists in patients before the period of marked general atherosclerosis or renal sclerosis a period during which the only demonstrable change in the body is an abnormal elevation of arterial pressure. This is the period to which Huchard has given the name "presclerosis." He has seen a number of large eaters, who may or may not have partaken of meat immoderately, in whom the arterial pressure did not surpass the normal figures, and a number of other subjects who presented arterial hypertension whose dietary habits could give rise to no criticism. On the contrary, he has often found among the immoderate users of alcoholic drinks an arterial hypertension which could be explained by no other cause. He attaches great importance to the studies of Aubertin, which show that the repeated ingestion of alcohol produces a hyperfunction of the suprarenal capsules in animals, absolutely parallel to that pointed out by Bernard and Bigert in lead poisoning. He does not mean to imply that the habitual and immoderate use of alcohol is the only cause of arterial hypertension; but he insists that arterial hypertension is one of the pathologic conditions which may follow this habit. Consequently it is necessary to regulate strictly the use of alcohol in all subjects who present arterial hypertension, particularly when the increased pressure can be explained in no other way. This is the only restriction he has to propose upon the diet of patients with hypertension. All other assertions about the quantity of foods absorbed, upon their nature, upon their greater or less content of salt have no acceptable clinical or experimental basis. There is a form of vascular sclerosis in which the changes attack only localized parts of the great vessels with the production of atheroma, usually not altering the small nutrient systems of the tissues or organs, and not accompanied by abnormal elevation of arterial pressure. This arteriosclerosis develops very gradually, and, as a rule, is observed only at an advanced age. In this form of arteriosclerosis no author has succeeded in establishing the noxious role of any food. In the dietary of these cases he advises the reduction in a moderate degree of those substances which we know may become harmful at a later period in the development of the disease: meals, which are small in amount, so as to prevent overfilling of the stomach; the moderate use of meat, red or white, as desired; less reduction of other foods except that their lime content should not be too high; abstinence from foods containing too great a proportion of salt with a recommendation to lessen the use of salt in the habitual dietary; the possibility of using light wines or beer, provided that the quantity of drink taken at each meal is moderate and to complete the quantity of fluid necessary in twenty-four hours by water taken between meals. If the disease is well advanced or if insufficiencies of the various organs begin to appear, a strict modication of diet is necessary. For kidney or liver insufficiency milk is necessary, without paying any attention to its high lime content. The amount of milk ordered will depend upon the gravity of the organic disorder. The complete suppression of meat should be prescribed only when there are signs of cardiorenal insufficiency. In the periods of improvement a small quantity of meat taken with the milk will prevent excessive emaciation. The legumes ought to form a large part of the diet of such a patient. The use of salt should be reduced as much as possible and fluids should be restricted to avoid the ingestion of too great amounts at one time. In cases of valvular disease of the heart one should not subject the patient to a too vigorous diet for fear of making existence insupportable. The patients should be allowed a liberal diet; but the amount taken at each meal should be small, and food which is difficult of digestion should be excluded, fats should be limited, the quantity of fluid taken at each meal ought to be limited, and the patients should give up alcoholic drinks and be very moderate in the use of tea and coffee. When decompensation begins, the dietary measures should be active. The three elements of diet that have been recognized as harmful in cardiac cases are salt, albuminoid substances, and fluids. In mild cases of decompensation salt should be excluded from the diet for two days every week or every two weeks. During the remaining part of the period the salt in the diet should not exceed two grams per day. The latter content is easily attained in a diet containing one liter of milk, a little meat, legumes, fruit and bread made without salt. In the more severe cases the exclusion of salt should be persisted in for one or two weeks at a time. As the symptoms improve salt may be added to the diet; but never more than three or four grams per day. It is of no benefit to restrict the use of meat absolutely in cardiac cases that are able to eliminate freely, such as well compensated valvular lesions and hypertension cases without renal involvement. However, as it is difficult to know exactly at what moment this elimination will become insufficient it is best to recommend that the dietary shall not contain too much meat. In cases of cardiac insufficiency without organic lesions of the kidneys, it is wise to reduce the total quantity of fluid and to have the fluid taken frequently in small doses. One will prevent in this way overloading the bloodvessels. In ambulant patients the fluid may be restricted in the day time but allowed freely in the evening, particularly when the patient is about to go to bed.

